# *Ubap1* knock-in mice reproduced the phenotype of SPG80

**DOI:** 10.1038/s10038-022-01073-6

**Published:** 2022-08-12

**Authors:** Keisuke Shimozono, Haitian Nan, Takanori Hata, Kozo Saito, Yeon-Jeong Kim, Hiroaki Nagatomo, Toshihisa Ohtsuka, Schuichi Koizumi, Yoshihisa Takiyama

**Affiliations:** 1 Department of Neurology, Graduate School of Medical Sciences, University of Yamanashi, Yamanashi, 409-3898 Japan; 2 Department of Neuropharmacology, Graduate School of Medical Sciences, University of Yamanashi, Yamanashi, 409-3898 Japan; 3 Department of Biochemistry, Graduate School of Medical Sciences, University of Yamanashi, Yamanashi, 409-3898 Japan; 4 Center for Life Science Research, University of Yamanashi, Yamanashi, 409-3898 Japan; 5 Department of Neurology, Fuefuki Central Hospital, Yamanashi, 406-0032 Japan

**Keywords:** Neurodegenerative diseases, Neurodegeneration

## Abstract

SPG80 is a neurodegenerative disorder characterized by a pure type of juvenile-onset hereditary spastic paraplegia and is caused by a heterozygous mutation of the UBAP1 (ubiquitin-associated protein 1) gene. UBAP1 is one of the subunits of the endosomal sorting complex required for transport I and plays a role in endosome sorting by binding to ubiquitin-tagged proteins. In this study, we generated novel *Ubap1*^+/E176Efx23^ knock-in mice, in which the SOUBA domain of Ubap1 was completely deleted with the UMA domain being intact, as an animal model of SPG80. The knock-in mice with this heterozygous *Ubap1* truncated mutation appeared normal at birth, but they developed progressive hind limb dysfunction several months later. Molecular pathologically, loss of neurons in the spinal cord and accumulation of ubiquitinated proteins were observed in *Ubap1*^+/E176Efx23^ knock-in mice. In addition, changes in the distributions of Rab5 and Rab7 in the spinal cord suggest that this mutation in *Ubap1* disturbs endosome-mediated vesicular trafficking. This is the first report of a mouse model that reproduces the phenotype of SPG80. Our knock-in mice may provide a clue for understanding the molecular pathogenesis underlying *UBAP1*-related HSP and screening of therapeutic agents.

## Introduction

Hereditary spastic paraplegias (HSPs) are a heterogeneous group of hereditary neurodegenerative disorders characterized by progressive weakness and spasticity of the lower extremities [1]. Although more than 80 genes or loci have been identified, the causative genes have not been identified in approximately 40% of HSP patients [2]. The clinical manifestations of HSP are classified into a pure form with leg spasticity as the main symptom and a complex one with other neurological symptoms [1–3].

SPG80, caused by *UBAP1* (ubiquitin-associated protein 1) mutations, is a pure form of juvenile-onset HSP reported recently [4–6]. UBAP1, which is one of the subunits of ESCRT-I, forms a stable complex with TSG101, VPS28, and VPS37A [7, 8]. *VPS37A* is the disease-causing gene of an autosomal recessive HSP (SPG53) [9]. Defects in TSG101, which is functionally related to UBAP1, have also been implicated in the development of neurological disorders including Charcot–Marie–Tooth disease type 1 C and spongiform neurodegeneration in mice, a prion-like disease [10–12]. ESCRTs are composed of four different protein complexes, ESCRT-0, I, II, and III. ESCRTs play a central role in intracellular trafficking, including the sorting of ubiquitinated proteins into endolysosomes and the formation of multivesicular bodies (MVBs) [13, 14]. In this process, ubiquitin-tagged proteins are transported to endosomes, where they fuse with lysosomes via MVB formation and are then degraded. This is an essential process for the destruction of misfolded or damaged proteins. Therefore, the dysfunction of ESCRTs is associated with neurodegenerative diseases.

In our previous study [6], we showed that our disease model with the UBAP1 C-terminal deletion (UBAP1-mutant) had lost its ubiquitin-binding domain and the ability to bind ubiquitin in vitro. We also found that the UBAP1-mutant might contribute to the disease phenotype via gain of function. However, further studies on animal models are warranted to elucidate the pathogenesis of *UBAP1* mutations in HSP.

Northern blot and reverse transcription-polymerase chain reaction (RT-PCR) analysis demonstrate a ubiquitous pattern of gene expression in a wide variety of human and mouse tissues, including the brain [15]. Undoubtedly, UBAP1 is involved in basic housekeeping functions of mammalian cells since it is preweaning lethal when deleted in mice (https://www.mousephenotype.org/data/genes/MGI:2149543). Therefore, we generated novel *Ubap1*-truncating mutation knock-in mice using CRISPR-Cas9 to examine whether they could reproduce the phenotype of SPG80. Moreover, we investigated the molecular pathology of the knock-in mice used in this study.

## Material and methods

### Animal experiments

Male C57BL/6 J mice obtained from SLC (Hamamatsu, Japan) were used in our experiments. Mice were maintained under standard conditions (24 ± 1 °C, 12 h light/dark cycle: lights on from 8 a.m. to 8 p.m.) with free access to food and water. Only male mice were used for the experimental analysis to avoid the effects of the estrous cycle and uncertain physiological conditions of female mice.

### Generation of *Ubap1* mutant mice using the CRISPR/Cas9 system

The *Ubap1* target site (5ʹ- GAGTTAAAAACCATTGATGA -3ʹ) was inserted into the bbsI site of the pX330-U6-Chimeric_BB-CBh-hSpCas9 plasmid (Addgene plasmid # 42230) for the synthesis of sgRNA. Then, the total length of the sgRNA was amplified by PCR with a primer to which a T7 site had been added, and the T7-sgRNA PCR product was used as the template for in vitro transcription using MEGAshortscript T7 (Thermo Fisher Scientific). The sgRNA was mixed with Alt-R S.p. Cas9 nuclease 3NLS (IDT), followed by incubation for 10 min at room temperature to yield an RNP complex with a final concentration of 20 ng/µl.

Pronuclear-stage B6D2F1 × C57BL/6 N zygotes generated by in vitro fertilization were used for microinjection. After microinjection, zygotes were incubated in mCZB medium at 37 °C until the next day under an atmosphere of 5% CO_2_ in the air. The 2-cell stage embryos were transferred into the oviducts of 0.5 d.p.c. pseudopregnant ICR females [16, 17]. The obtained pups were genotyped by PCR and subsequent Sanger sequencing. To avoid plausible off-target mutations, five generations of backcrossing were performed.

For genotyping, genomic DNA was isolated from tissue obtained from the tip of the tail or from an ear using standard procedures. Genomic DNA was amplified with the following primers: 5ʹ- CAAAGTGAGCTTCCCCAAGACT -3ʹ (forward) and 5ʹ- GCAGGATGTGCTATGGAAAGTG -3ʹ (reverse).

### Western blot analysis

Western blotting was performed using an enhanced chemiluminescence system. Immunoblots were obtained for total protein extracts of the brains of 7-month-old wild-type (*N* = 3) and heterozygous *Ubap1*^+/E176Efx23^ knock-in mice (*N* = 3). Briefly, the proteins were harvested from brain tissue by homogenization in RIPA buffer with a protease inhibitor cocktail (Roche). Equal amounts of proteins were separated on 10% SDS–polyacrylamide gels and then electrotransferred onto polyvinylidene difluoride membranes. After blocking with 3% BSA in PBS, the membranes were incubated with a primary antibody. After washing with PBS-0.1% Tween 20, filters were probed with horseradish peroxidase-conjugated sheep anti-rabbit IgG or rabbit anti-mouse IgG (Cell Signaling; Beverly, MA, USA). Immunoreactivity was detected with an enhanced chemiluminescence system (Amersham Biosciences, Buckinghamshire, UK). The chemiluminescent signals were captured with a Fujifilm luminescent image LAS-4000 analyzer (Fujifilm, Tokyo, Japan). Two kinds of rabbit polyclonal anti-UBAP1 antibodies (Sigma-Aldrich, SAB1307218 and Invitrogen, PA5-49644) were used in this study.

### Reverse transcription polymerase chain reaction (RT-PCR) and Sanger sequencing

Total RNA from brain tissue was extracted using Trizol (Invitrogen) according to the manufacturer’s instructions. Reverse transcription was performed using a Bio-Rad iScript™ gDNA Clear cDNA Synthesis Kit. Primers were used to amplify the mutation site including exon 4, exon 5, and the flanking intronic region: 5ʹ-CTGACTTCGAGTGTGAAGAGGACC-3ʹ (forward) and 5ʹ-ATGTTGGGCACTTGTGACACTG-3ʹ (reverse). PCR was performed with KAPA Taq (Kapa Biosystems), and the PCR products were purified with an Illustra™ ExoProStar™ purification kit (Cytiva™). Sanger sequencing was performed by Eurofins Genomics, and trace files were analyzed with Sequence Scanner Software.

### Restriction fragment length polymorphism (RFLP) analysis of the heterozygous *Ubap1* truncated mutation

Extraction of total RNA and cDNA synthesis were described as above. A 91-bp fragment encompassing the heterozygous *Ubap1* truncated mutation was amplified using mismatched forward and reverse primers with the following sequences: 5ʹ-TGACTTCGAGTGTGAAGTGGACCCATTTGA-3ʹ (forward) and 5ʹ-GTTCCTACCAGAATGTTTCTCAGCACTTCCTTC-3ʹ (reverse). PCR was performed with EmeraldAmp PCR Master Mix (Takara Bio), and the PCR products were purified with an QIAquick PCR Purification Kit (Qiagen). Primer mismatching eliminates the MboII restriction site near the mutation site in the mutant polymerase chain reaction (PCR) product. The product was then digested with the restriction enzyme MboII (NEB, R0148S).

### Behavioral studies

In the beam-walk assay, from 2 to 6-month-old male mice were made to walk on a bar placed parallel to the ground at a height of 70 cm. The bars were made of stainless steel, and had a diameter of 1 cm and a length of 100 cm. To quantify the gait disturbance, the number of slips from the beam was determined. Three walking tests were performed and the average number of slips was recorded, i.e., 10 for falling from the beam, and the maximum number of slips per test was also set to 10. In addition, we measured the foot-base-angle (FBA) at toe-off positions of the hind-paws using video recordings of beam-walking mice. The left and right foot-base-angles were measured twice each, and the lower value of the average was recorded.

### Tissue preparation and immunofluorescent staining

Mice were anesthetized with three types of anesthetic agents (medetomidine, midazolam, and butorphanol) via intraperitoneal administration. The mice were anesthetized and perfused with ice-cold phosphate-buffered saline (PBS, 0.1 M, pH 7.4) transcardially, followed by 4% paraformaldehyde (PFA) in PBS. After perfusion, their brains and spinal cords were removed, post-fixed in 4% PFA overnight at 4 °C, and then cryoprotected in a 30% w/v sucrose solution for two days. The frozen brains and spinal cords were sectioned at 20 μm thickness, using a cryostat (CM1520, Leica). The sections were washed 3 × 5 min in PBS, blocked with 0.1% Triton X-100/10% NGS for 1 h at room temperature, and then incubated with primary antibodies for two days at 4 °C. The following primary antibodies were used, rabbit anti-Rab5 (1:200; Thermo Fisher), rabbit anti-Rab7 (1:200; Thermo Fisher), rabbit anti-UBAP1 (1:1000; Sigma-Aldrich), rat anti-GFAP (1:500; Thermo Fisher), rabbit anti-NeuN (1:500; Millipore), mouse anti-Ubiquitin (1:500; Cell Signaling), rabbit anti-LC3 (1:300; Cell Signaling), and mouse anti-MAP2 (1:500; Sigma-Aldrich). After conjugation with the primary antibodies, the sections were washed 3 × 5 min in PBS and then incubated with the corresponding secondary antibodies (1:1000; Thermo Fisher) for 2 h at room temperature. The sections were then washed 3 × 5 min in PBS and fluorescence images were obtained using a confocal laser microscope system (FV-1200; Olympus). For microscopic examination, spinal cords were harvested in the same manner as above. 1-μm sections from wild-type and *Ubap1* knock-in mice were stained with toluidine blue to identify axons in cross-sections. Sections of the lumbar region were used for analysis. Images were processed with Image J.

### Nearest neighbor analysis

We performed nearest neighbor analysis to quantify the distributions of Rab5 and Rab7 puncta. The nearest neighbor formula produces a result between 0 and 2.15, where 0 indicates a clustered, 1 a random, and 2.15 a regular distribution.

The formula used is as follows:

$$Rn = \frac{{\bar D(Obs)}}{{0.5\sqrt {\frac{a}{n}} }}$$

(Rn: nearest neighbor value, ${{{\bar{\mathrm D}}}}$ (Obs): mean observed nearest neighbor distance, a: area under study, n: total number of points).

### Statistical analysis

Data are presented as means ± S.D. and box plots show the distributions of the data. The middle line indicates the median and the upper and lower whiskers indicate the maximum and minimum data. Statistical analysis was performed using Welch’s *t*-test assuming unequal variance, and *p* < 0.05 was considered to be significant. The Mann–Whitney *U* test was used for comparison between two groups.

## Results

### Generation of *Ubap1* knock-in mice

**Fig. 1:**
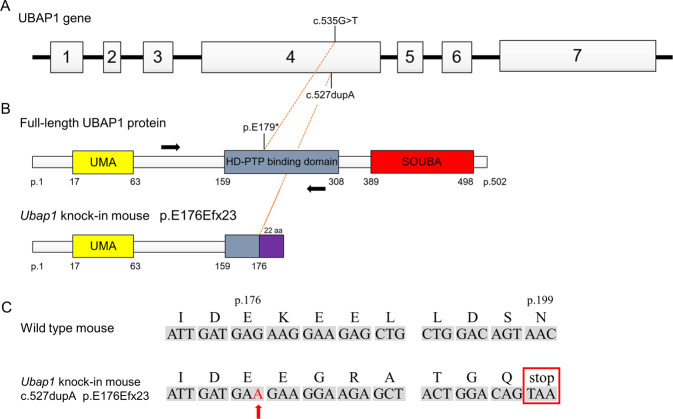
**A**, **B** UBAP1 gene and protein structure, and predicted effects on the protein structure of the mutation in our UBAP1+/E176Efx23 knock-in mouse model. The locations of the mutations in our patient (c.535 G > T, p.E179*) and the model mouse (c.527dupA, p.E176Efx23) we created are shown. The locations of the PCR primers used to screen for the presence of the targeted mutation are salso hown (black arrow). **C** Sanger sequencing results for wild-type mice and *Ubap1*^+/E176Efx23^ knock-in mice. The mutation of c.527dupA (red arrow) was detected in *Ubap1*^+/E176Efx23^ knock-in mice, while no mutation was detected in wild-type mice

UBAP1 comprises a 502 amino acid residue protein that is widely expressed in human tissues and broadly conserved among species, and human UBAP1 shows more than 90% sequence identity to the mouse Ubap1 protein. Mammalian UBAP1 consists of three major domains: the N-terminal UMA (UBAP1-MVB12-associated) domain, the HD-PTP (HIS-domain protein tyrosine phosphatase) binding region, and the C-terminal SOUBA (solenoid of overlapping ubiquitin-associated) domain [8] (Fig. 1B). The mutations of *UBAP1* in the four families we previously reported as well as the other families with SPG80 reported from all over the world so far all result in prematurely truncated proteins with complete loss of the C-terminal SOUBA domain, the N-terminal UMA domain remaining intact [4–6, 18–20]. Therefore, we generated knock-in mice in which the SOUBA domain was completely deleted with the UMA domain being unaffected. In accordance with the four families reported previously [6], we generated mutant mice genetically similar as to the mutation (c.535 G > T, p.E179*) that resulted in the SPG80 phenotype. The mutation in the knock-in mice obtained was c.527dupA (p.E176Efx23), which would theoretically cause the same dysfunction (Fig. 1A, B, C). However, it is noteworthy that CRISPR can induce off-target editing at genomic positions that imperfectly match the sgRNA sequence. Therefore, given the possibility that there are genomic regions of close homology to the sgRNAs used for Cas9-dependent cleavage, the possibility that these off-target sites are also modified should be considered. We screened off-target candidates from the whole mouse genome sequence using CRISPRdirect (https://crispr.dbcls.jp/) (Supplementary Table 1). Examination of the mouse genome DNA with 20-nucleotide gRNA sequences revealed no 15 or more nucleotide matches, but three sequences with 14-nucleotide matches. These three off-target candidates were examined by direct sequencing analysis. No mutations were found in any of the off-target candidates, indicating that the chance that Cas9-mediated off-target mutation had occurred was low.

*Ubap1*^+/E176Efx23^ knock-in mice were delivered in expected Mendelian ratios and were viable with normal reproduction. However, none of the *Ubap1*^E176Efx23/E176Efx23^ mice were born, which is consistent with previous finding that *Ubap1* is expressed in a wide range of tissues, and its loss in homozygous mice is embryonic lethal [7].

### mRNA expression of *UBAP1* mutant allele in brain

**Fig. 2:**
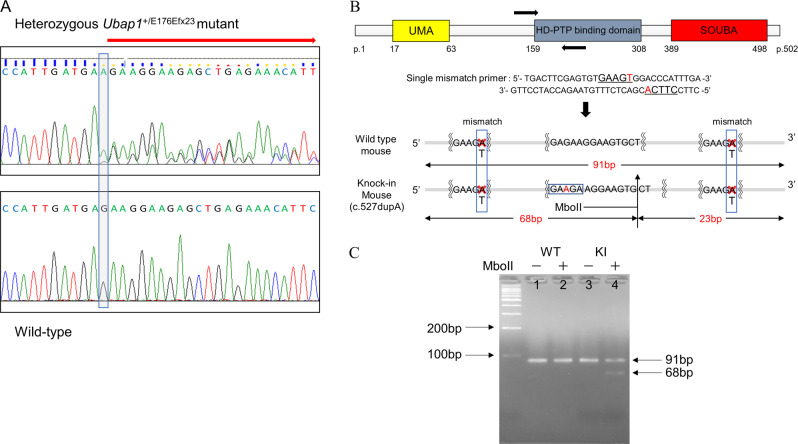
**A** RT-PCR of brain tissue from wild-type and heterozygous *Ubap1*^+/E176Efx23^ mutant knock-in mice at 7 months of age indicates that truncated UBAP1 mRNA escapes nonsense-mediated decay in mutant mice brains. The boted area indicates the location of c.527dupA. **B** Schematic representation of RFLP analysis. Primers used in RT-PCR and their positions are indicated by arrows (upper). This site shows the expected restriction fragments following digestion with MboII. **C** Electrophoresis pattern of RFLP. Lanes 1, 2, and 3 shows the 91 bp fragment, and lane 4 shows both the 91 bp and 68 bp fragments (fragment 23 not visualized)

To determine whether the observed truncating variants would lead to nonsense-mediated mRNA decay in mouse brains, we evaluated the mRNA expression of the mutant allele. RT-PCR was performed on RNA extracted from the brain tissue from the wild-type and heterozygous *Ubap1*^+/E176Efx23^ mutant knock-in mice, and the RNA was sequenced by the Sanger method. Surprisingly, the heterozygous c.527dupA p.E176Efx23 mutant transcript was detected in the mutant mice cDNA, indicating escape from nonsense-mediated mRNA decay (Fig. 2A). To confirm the ratio between wild-type and mutant mRNA, we performed RFLP analysis on mice brain cDNA. The amplified cDNA from the heterozygous *Ubap1*^+/E176Efx23^ mutant was digested with MboII (Fig. 2B). We observed two fragments of 91 bp and 68 bp (23 bp fragment not visible), indicating the mutant mRNA was expressed in the brain (Fig. 2C). The intensity of the observed fragments is approximately half that of the fragments observed in the knock-in mice without the restriction enzyme, indicating that the mutant mRNA is normally expressed.

We next performed immunoblot analysis to evaluate both the wild-type and potential truncated mutant Ubap1. Although we thoroughly validated several antibodies raised against the N-terminal region of mouse Ubap1, unfortunately, we failed to detect endogenous Ubap1 in the mouse brain. For this reason, we prepared a plasmid expressing the N-terminally Flag epitope-tagged Ubap1 mutant, and then validated its expression in Neuro2a cells by transfection. After transfection, we successfully detected both Ubap1 wild-type and Ubap1 mutant expression in Neuro2a cells on Western blotting (Supplementary Fig. 1A). This suggests the mutant protein could be normally expressed.

### *Ubap1*^+/E176Efx23^ knock-in mice developed progressive spastic gait

**Fig. 3:**
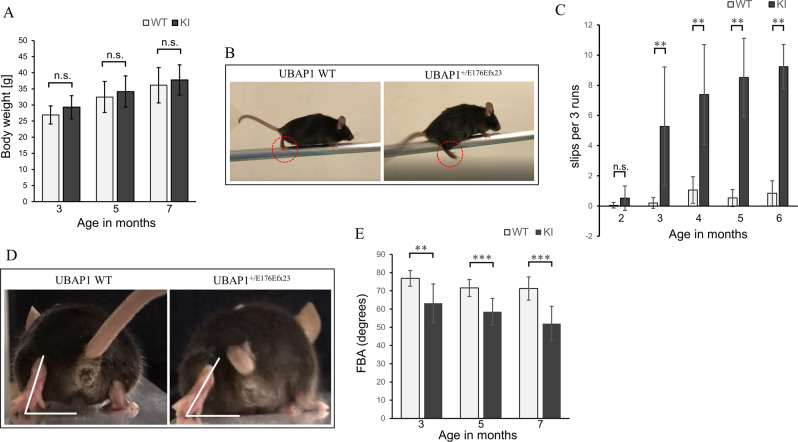
*Ubap1* knock-in mice developed progressive spastic gait. **A** There was no difference in body weight between the genotypes at 3, 5, and 7 months of age (WT: *N* = 16, KI: *N* = 13; Welch’s *t*-test; n.s. not significant). **B** Representative photos of wild-type and *UBAP1*^+/E176Efx23^ knock-in mice walking on a beam, taken from the side. *Ubap1*^+/E176Efx23^ knock-in mice show a marked decrease in the ability to grasp the beam with their hind limbs (compare the dashed circles for the two genotypes). **C** *Ubap1*^+/E176Efx23^ knock-in mice showed an apparent age-dependent increase in the number of slips on the beam (WT: *N* = 16, KI: *N* = 13; Mann–Whitney *U* test; n.s. not significant, **: *p* < 0.01). **D** The foot-base-angle (FBA) at the toe-off position of the hind leg is indicated by the white line (7 months old). **E** There is a significant age-dependent decrease in the FBA of *Ubap1*^+/E176Efx23^ knock-in mice (WT: *N* = 13, KI: *N* = 15; Welch’s *t*-test; **p < 0.01, ***p < 0.001). Results are expressed as means ± SD

*Ubap1*^+/E176Efx23^ knock-in mice appeared normal at birth and showed no obvious difference in body weight compared to the wild-type (Fig. 3A). At the age of two months, there were no significant differences in the numbers of slips and falls between *Ubap1*^+/E176Efx23^ knock-in mice and *Ubap1* wild-type mice. However, at the age of three months, the numbers of slips and falls were significantly increased in *Ubap1*^+/E176Efx23^ knock-in mice. On the other hand, the wild-type mice showed fewer slips and falls during the test (Fig. 3B, C). These increases in frequency were thought to be correlated with decreases in motor coordination of the mutant mice. They also showed a marked decrease in the ability to grasp sticks with the hind limbs, which is similar to the phenotype reported for other HSP mouse models (Fig. 3B) [21]. At 6 months of age, they had difficulty crossing a beam and showed significant differences in gait from wild-type mice (Supplementary Videos 1 and 2).

At 3 months of age, there was also a significant decrease in the FBA compared to in the wild-type (Fig. 3D, E). At three months, the FBA had decreased to about 65 degrees in mutant mice, and then it decreased progressively with growth to about 50 degrees at six months. The same type of gait disturbance was observed in female mice as well as male ones. No gender differences in human SPG80 patients were found in the previous studies [4–6, 18–20], which is consistent with the results of our animal study. No physical change other than the gait disturbance was observed, and the combination of hindlimb muscle weakness and spasticity closely resembled the clinical symptoms of human HSP patients, suggesting that *Ubap1*^+/E176Efx23^ knock-in mice can be a clinically valid disease model for SPG80.

### Mutations in *Ubap1* result in axonopathy of the central nervous system

**Fig. 4:**
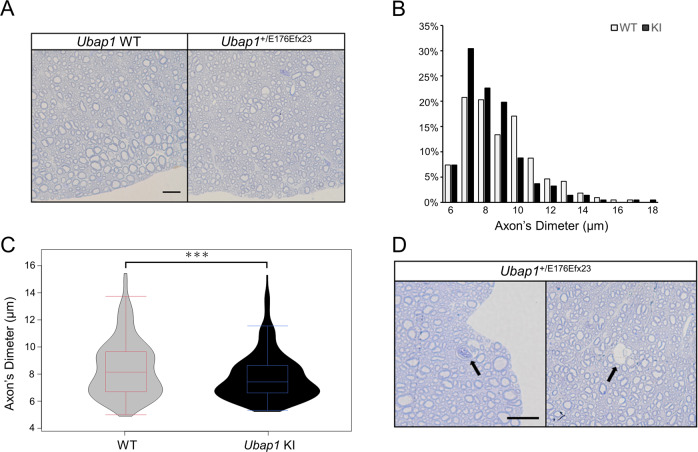
Heterozygous deletion of Ubap1 results in axonopathy of the central nervous system. **A** Semithin sections of the corticospinal tract in the spinal cords of 7-months-old mice at the lumbar level. Scale bar, 20 μm. **B** The sizes of axons were determined and the percentage of each size is shown. **C** The violin plots show that thick myelinated fibers are selectively lost in *Ubap1*^+/E176Efx23^ knock-in mice (*n* = 216 each; Mann–Whitney *U* test; ***p < 0.001). **D** Arrows denote direct evidence of axonal degeneration, which was not observed in the wild-type mice. Scale bar, 20 μm

Axonal degeneration has been reported as the main pathological change in other pure-type HSP mouse models, such as SPG4 and SPG31 [22, 23]. Therefore, we performed toluidine blue staining of transverse semi-thin sections of the spinal cord of *Ubap1*^+/E176Efx23^ mice at seven months of age to observe axons. The characteristic change in *Ubap1*^+/E176Efx23^ knock-in mice was the selective loss of thick myelinated fibers close to the spinal cord surface (Fig. 4A). In wild-type mice, myelinated fibers distributed bimodally with diameters of approximately 7 μm and 10 μm were most abundant, whereas in *Ubap1*^+/E176Efx23^ knock-in mice, myelinated fibers were destributed unimodally, with as small as 7 μm fibers being most abundant (Fig. 4B, C), which is indicative of axonal degeneration. We also observed direct evidence of axonal degeneration, which has also been observed in other pure-type HSP mouse models (Fig. 4D).

### Mutations in *Ubap1* affect the distributions of Rab5 and Rab7

ESCRT-I plays a role in the recognition of ubiquitinated cargoes in endosomes and the transport of these cargoes to late endosomes to form MVBs [24]. Because the UBAP1 protein functions in the early endosomal compartment of neurons [18], brain and spinal cord sections from 7-month-old mice of both genotypes were collected by the same method as that described above, immunostained with Rab5 and Rab7, and then photographed under confocal microscopy. Rab5 is localized to the early endosomes, which regulates the vesicular trafficking and the fusion of early endosomes during endocytosis [25]. Rab7 is localized to late endosomes and lysosomes, where it regulates vesicular trafficking and membrane fusion processes [26, 27].

**Fig. 5:**
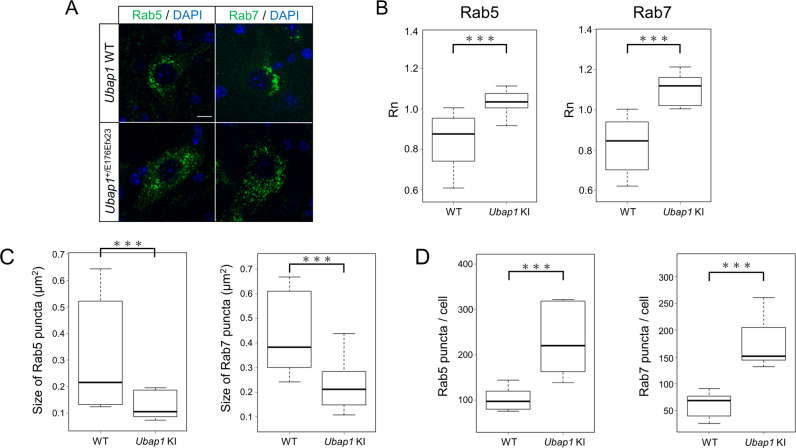
Mutations in *Ubap1* affect the distributions of Rab5 and Rab7. **A** Confocal images of spinal cord sections from 7-month-old mice stained for Rab5 (green), Rab7 (green), and DAPI (blue). Scale bar, 10 μm. **B** Nearest neighbor analysis was performed to quantify the distributions of Rab5 and Rab7 puncta, which are shown in the graph (Rab5: *n* = 13, Rab7: *n* = 12; Mann–Whitney *U* test; ***: *p* < 0.001). **C**, **D** Quantification of the number and size of Rab5 and Rab7 positive puncta was performed (Rab5: *n* = 13, Rab7: *n* = 12; Mann–Whitney *U* test; ***: *p* < 0.001)

Most Rab5 and Rab7-positive endosomes are constant in size, and are distributed near the plasma membrane and around the nucleus, respectively, [28, 29]. In our study, both the two markers (Rab5 and Rab7) showed similar fluorescence patterns for each genotype, and the results were reproducible. In wild-type mice, they were localized around the nucleus, and in *Ubap1*^+/E176Efx23^ knock-in mice, they were diffuse in the neurons (Fig. 5A). To quantify these findings, we performed nearest neighbor analysis and confirmed that there is a difference in distribution between the wild-type and *Ubap1*^+/E176Efx23^ knock-in mice (Fig. 5B).

In addition to this distribution pattern change, *Ubap1*^+/E176Efx23^ knock-in mice showed smaller endosomes and an increase in their vesicle number (Fig. 5C, D). Alterations in the structure and distribution of Rab proteins are closely related to disease onset and pathogenesis, suggesting that the abnormal endosome formation and fusion observed in *Ubap1*^+/E176Efx23^ knock-in mice would lead to impaired vesicle traffic, which contributes to the disease pathogenesis [30–32].

### Mutation of *Ubap1* results in accumulation of ubiquitinated proteins and neuron loss in the spinal cord

**Fig. 6:**
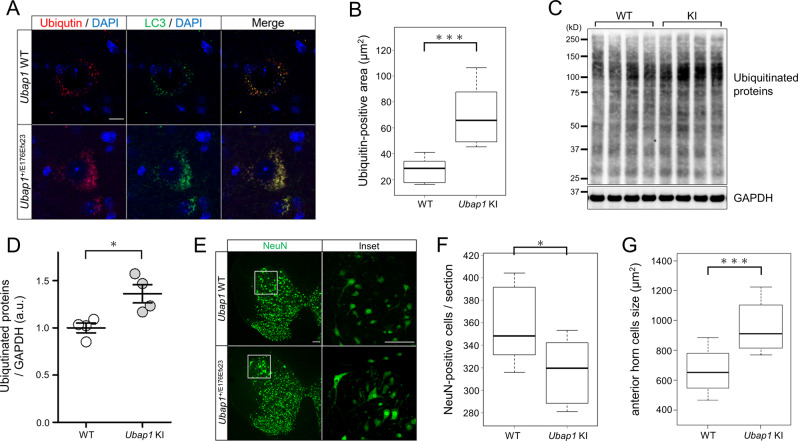
Mutation of *Ubap1* results in the accumulation of ubiquitinated proteins and loss of neurons in the spinal cord. **A** Confocal images of spinal cord sections from 7-month-old mice stained with ubiquitin (red), LC3 (green), and DAPI (blue). Yellow in the merged images indicates colocalization of ubiquitin and LC3. Scale bar, 10 μm. **B** Comparison of the area stained with ubiquitin in a single neuron is shown in the graph (WT: *n* = 28, KI: *n* = 30; Mann–Whitney *U* test; ***: *p* < 0.001). **C**, **D** Quantification of ubiquitinated proteins from mouse spinal cord tissue by Western blotting. The expression of GAPDH is shown as a loading control. Mutations in *Ubap1* result in increases in the ubiquitinated proteins (WT: *N* = 4, KI: *N* = 4; Welch’s *t*-test; *: *p* < 0.05). **E** Confocal images of spinal cord sections from 7-month-old mice stained for NeuN (green). The image on the right is a higher magnification of the area indicated by the square. Scale bar, 100 μm. **F** Quantification was performed. Mutations in *UBAP1* reduced the number of neurons in the spinal cord (WT: *n* = 14, KI: *n* = 11; Mann–Whitney *U* test; *: *p* < 0.05). **G** Mutations in *Ubap1* caused an increase in the size of the anterior horn cells of the spinal cord (WT: *n* = 55, KI: *n* = 42; Mann–Whitney *U* test; ***: *p* < 0.001)

To assess the effects of the *Ubap1* mutant on the central nervous system, 7-month-old wild-type mice (*N* = 4) and *Ubap1*^+/E176Efx23^ knock-in mice (*N* = 3) were perfused with paraformaldehyde, and then their brains and spinal cords were extracted. UBAP1 is one of the subunits of ESCRT-I and plays a role in the sorting of ubiquitinated cargoes by binding to ubiquitin via its SOUBA domain [8]. Since the UBAP1-mutant has lost its ability to bind ubiquitin, it is expected that the sorting of ubiquitinated cargoes is severely affected. Therefore, we examined ubiquitin and autophagosome LC3 in neurons of the lumbar spinal cord by immunostaining. Compared within wild-type mice, the drastic accumulation of ubiquitinated proteins was observed in *Ubap1*^+/E176Efx23^ knock-in mice (Fig. 6A, B). In addition, autophagosome accumulation co-localized with the aggregation of ubiquitinated proteins was detected (Fig. 6A), indicating dysfunction of autophagy and the endosomal-lysosomal pathway. Accumulation of ubiquitinated proteins was also observed on Western blotting of proteins extracted from brains of *Ubap1*^+/E176Efx23^ knock-in mice (Fig. 6C, D), and Neuro2a cells with exogenous expression of Flag-UBAP1E176Efx23 (Supplementary Fig. 1B, C).

To assess the effects of the Ubap1 mutant on neuron degeneration, sections of the spinal cords were labeled by NeuN-immunostaining. The results showed that the number of neurons in the spinal cord of *Ubap1*^+/E176Efx23^ knock-in mice was reduced compared to in the wild-type mice at seven months of age (Fig. 6E, F). Notably, there was apparent hypertrophy of the anterior horn cells (Fig. 6E, G). Dysfunction of autophagy or the endosomal-lysosomal pathway might result in neuronal hypertrophy and a decrease in the number of neurons.

## Discussion

Although SPG80 is known to be associated with *UBAP1* mutations, the molecular and cellular mechanisms underlying SPG80 remain unknown. An animal model for SPG80, an *UBAP1* knock-down zebrafish, has been shown to have significantly shorter motoneuron axon lengths than those in wild-type ones. There was also decreased mobility and a shortened lifespan, indicating that UBAP1 may contribute to motor development and normal behavior [4, 5].

However, to our knowledge, no mouse model has been reported to reproduce the phenotype of SPG80. The knockout mouse models reported so far completely lack the entire functional domain of Ubap1, including not only the SOUBA domain but also the UMA domain that binds to ESCRT-I [5]. In our mouse model, although the SOUBA domain was completely deleted, the UMA domain was completely preserved. Theoretically, the binding to the ESCRT-I complex would be preserved. It is thus possible that the expression of the UMA domain alone may be crucial and toxic. Our study indicates that the expression of the UMA domain alone might reproduce the SPG80 phenotype due to arrest of the ESCRT-complex without the acquisition of ubiquitinated protein cargo through a gain of function mechanism.

*Ubap1*^+/E176Efx23^ knock-in mice developed progressive hindlimb dyskinesia from an early stage. There were no physical changes other than the gait disturbance, which was limited to the upper motor neurons. These findings are consistent with human SPG80. Thus, we conclude that our mouse model is valid for studying the pathophysiology of human SPG80.

Pathologically, there was a morphological difference between Rab5 and Rab7. In *Ubap1*^+/E176Efx23^ knock-in mice, in addition to changes in the distributions of Rab5- and Rab7-positive endosomes, increases in their numbers and reductions in their sizes were observed. This finding suggests that mutations in *UBAP1* may impair endosome fusion and maturation, leading to impaired vesicular trafficking. In order for unwanted proteins to be degraded through the endosome-lysosome pathway, early endosomes need to mature and fuse with lysosomes. However, the degradation of these proteins may be partially hindered by the inhibition of endosome fusion and maturation, and the concomitant impairment of vesicle traffic [33]. The accumulation of ubiquitinated proteins and autophagosomes, the enlargement of anterior horn cells, and the decrease in the number of neurons may be due to the endosome dysfunctions.

Endosome maturation has also been shown to be regulated by the protrudin-PDZD8 complex. The gene encoding for protrudin *(ZFYVE27)* is known to be the causative gene for SPG33 [34]. Furthermore, protrudin interacts with other HSP-related proteins such as SPG2, SPG3A, SPG31, and SPG10, suggesting that inhibition of endosome maturation is associated with the development of other HSPs [35, 36].

The neuronal loss observed in our mouse model has been reported previously in an in vitro study, suggesting the involvement of apoptosis as the disease mechanism [5]. However, neuronal loss and morphological changes in the motor cortex and spinal cord have not been reported in SPG4 and SPG31 mouse models. The main pathological change in other pure-type HSP mouse models was axonal degeneration, which leads to impaired axonal transport [22, 23]. Axonal degeneration is one of the most common mechanisms underlying HSPs, which was also observed in our mouse model. In the superficial layer of the lumbar spinal cord, selective loss of some thick myelinated fibers was observed. In many neurodegenerative diseases, axonal degeneration precedes cell body death, and this axonal degeneration is thought to be an early step in neuron-wide degeneration.

RFLP analysis revealed that the mutant Ubap1 mRNA (p.E176Efx23) was normally expressed in the brain. This indicates nonsense-mediated mRNA decay does not occur in the mutant mice. In addition, we showed the truncated UBAP1 protein expression in Neuro2a cells. Taken together, these results suggest that both mRNA and protein expression of the UBAP1 mutant allele are normal, which supports the possibility that SPG80 is caused by toxicity or gain of function due to UBAP1 mutations.

In summary, we generated the first *Ubap1* knock-in mice that reproduce the phenotype of SPG80 and showed the dysfunction of endosomes in the *Ubap1* knock-in mice. *Ubap1* mutations may impair endosome fusion and maturation, leading to impaired vesicle traffic. Disruption of endosome fusion and maturation may inhibit the subsequent endosome-lysosome degradation pathway, leading to the accumulation of unwanted ubiquitinated proteins, axonal degeneration, and even neuronal loss. Our knock-in mice may provide a clue for further understanding the molecular pathogenesis underlying *UBAP1*-related HSP as well as screening for the therapeutic agents in future.

## Supplementary information

supplementary figure

supplementary table

supplementary video 1

supplementary video 2

Supplementary fig. and videos caption
