# Supplementary material for: Ubap1 knock-in mice reproduced the phenotype of SPG80
Source: J Hum Genet. 2022 Aug 12;67(12):679–86. doi: 10.1038/s10038-022-01073-6 (PMC9691459; doi:10.1038/s10038-022-01073-6)
Supplement: Supplementary file 2 — supplementary table [file 10038_2022_1073_MOESM2_ESM.docx]

Table S1. Predicted off-target sites

*Nucleotide matched sgRNAs are highlighted in red.
