# Supplementary material for: Ubap1 knock-in mice reproduced the phenotype of SPG80
Source: J Hum Genet. 2022 Aug 12;67(12):679–86. doi: 10.1038/s10038-022-01073-6 (PMC9691459; doi:10.1038/s10038-022-01073-6)
Supplement: Supplementary file 5 — Supplementary fig. and videos caption [file 10038_2022_1073_MOESM5_ESM.docx]

**Supplementary Video 1.** A 4.5-month-old *Ubap1*^+/E176Efx23^ knock-in mouse in a beam-walking test.

**Supplementary Video 2.** A 4.5-month-old *Ubap1* wild-type mouse in a beam-walking test.

**Supplementary Figure 1.**

A. Protein expression of UBAP1 and UBAP1 mutants in Neuro2a cells.

B-C. Quantification of ubiquitinated proteins in Neuro2a cells expressing the UBAP1 mutant. The exogenous expression of Flag-UBAP1E176Efx23 would cause the accumulation of ubiquitinated proteins in Neuro2a cells (WT: n= 4, KI: n=4; Welch’s t-test; *: p<0.05).
